# Validation of the Persian version of the 40-item amyotrophic lateral sclerosis assessment questionnaire

**Published:** 2013

**Authors:** Hosein Shamshiri, Mohammad Reza Eshraghian, Nastaran Ameli, Shahriar Nafissi

**Affiliations:** 1 Resident, Department of Neurology, Shariati Hospital, Tehran University of Medical Sciences, Tehran, Iran; 2 Professor, Department of Statistics and Epidemiology, School of Health, Tehran University of Medical Sciences, Tehran, Iran; 3 Department of Biostatistics and Epidemiology, Tehran University of Medical Sciences, Tehran, Iran; 4 Associate professor, Department of Neurology, Shariati Hospital, Tehran University of Medical Sciences, Tehran, Iran

**Keywords:** 40-Item Amyotrophic Lateral Sclerosis Assessment Questionnaire, Cronbach's Alpha Coefficient, Item-Total Correlation, SF-36 Questionnaire, Amyotrophic Lateral Sclerosis Functional Rating Scale-revised

## Abstract

**Background:**

As a disease of motor nervous system (motor neuron disease), amyotrophic lateral sclerosis (ALS) has a great impact on several aspects of quality of life (QoL). Generic questionnaires of QoL do not address all the especial features of ALS and therefore translation and validation of disease specific questionnaires such as Amyotrophic Lateral Sclerosis Assessment Questionnaire 40-item (ALSAQ-40) is necessary for assessment of patients with different languages. The aim of this study was to review the validation of the Persian version of the ALSAQ-40.

**Methods:**

Meticulously translated ALSAQ-40 was completed by 21 ALS patients. Internal reliability was evaluated using Cronbach's alpha coefficient and item-total correlation was also used to evaluate the correlation of each question with total score. Validity was evaluated through comparison with Amyotrophic Lateral Sclerosis Functional Rating Scale-revised (ALSFRS-r) and the 36-Item Short Form Health Survey (SF-36).

**Results:**

Cronbach's alpha coefficient was 0.91-0.96 for different scales of the ALSAQ-40. All the 40 questions of the questionnaire had correlation greater than 0.5. Correlation coefficient of all the related scales of the Persian version of ALSAQ-40, SF-36 and ALSFRS-r was greater than 0.59 with P value < 0.001.

**Conclusion:**

Measures of the Cronbach's alpha coefficient and item-total correlation demonstrated reliability and consistency of the questionnaire, and correlation coefficients confirmed the validity of different items in the questionnaire. This study showed that the Persian version of the ALSAQ-40 is a reliable and valid questionnaire for the evaluation of QoL in ALS patients with Persian language.

## Introduction

Amyotrophic lateral sclerosis (ALS) is a fatal neurodegenerative disease which involves the motor system. As the disease progresses, patients become progressively paralyzed and more dependent on caregivers even for minor activities of daily living. Besides, swallowing and respiratory impairment and dysarthria aggravate patient's condition. The pathogenesis of ALS is not well understood and there is no cure for this disease. Therefore, nowadays the main focus is on improving the quality of life (QoL) during the disease span.^1–4^ QoL is how an individual perceives the situation in the context of cultural and societal values and it is related to patients’ objectives, expectations, standards and interests. Several questionnaires have been developed in order to evaluate this item which have been translated in multiple languages versions.^5, 6^

The 40-Item Amyotrophic Lateral Sclerosis Assessment Questionnaire (ALSAQ-40) is specifically designed to evaluate the QoL of ALS patients. Forty questions each with five choices constitute this questionnaire from which 10 questions are about patient's physical aspects and mobility, 10 are about activities in daily living and independence, 3 are about eating and drinking, 7 are about communication and 10 are about emotional aspects. ALSAQ-40 asks about the patient's condition in two recent weeks. ALSAQ-40 was designed in 1999 by Jenkinson et al. and its internal reliability was evaluated using Cronbach's alpha coefficient in two separate studies. It was demonstrated that Cronbach's alpha coefficient in all the five domains was greater than 0.9 which means good internal reliability. Besides, validity of the ALSAQ-40 was evaluated through correlation of each domain with the related domain of SF-36 and ALSFRS-r; correlations were significant which confirmed the validity of the ALSAQ-40.^7^

ALSAQ-40 is now used in several countries to evaluate several aspects of the health, QoL and impact of managements in ALS patients.^5, 7–14^ The present study aimed to evaluate the internal reliability and validity of Persian version of ALSAQ-40 and its scales and subscales.

## Materials and Methods

The ALSAQ-40 was translated to Persian by two physicians and then was evaluated and edited by a neurologist and was retranslated (back-translation) to English by two other independent translators. The back-translation was compared with the original manuscript and the ultimate Persian version was prepared.

Twenty-one patients of both genders were enrolled with definite and laboratory supported probable motor neuron impairment according to El Escorial criteria. The patients completed the ALSAQ-40 and SF-36 questionnaires, and the ALSFRS-r was completed by a physician.

The SF-36 is a generic questionnaire which was translated and validated in Persian and is used for evaluating the QoL in ALS patients.^6^ This questionnaire is consisted of 36 questions in eight domains including four physical components (physical functioning, role limitations due to physical health, bodily pain, and general health perceptions) and four mental components (vitality, social functioning, role limitations due to emotional problems, and mental health). The final score is between 0 and 100 and higher score means better QoL.^8^

Amyotrophic Lateral Sclerosis Functional Rating Scale-revised (ALSFRS-r) is a scoring system which is consisted of 12 subscales in four domains including bulbar function, fine motor function, gross motor function and respiratory function. Each subscale is scored 0 to 4 and the total score is between 0 and 48; higher score means less functional disability.^9^

Internal reliability was evaluated using Cronbach's alpha coefficient which greater than 0.7, indicates high internal consistency of every subscale's question. Then the consistency of each question with total score subscale was evaluated using item-total correlation; and measures greater than 0.4 indicated a satisfactory correlation.

Validity was evaluated using SF-36 questionnaire for assessment of the physical and mental aspects, and ALSFRS-r for assessment of other aspects of the Persian version of the ALSAQ-40. Since high score in SF-36 questionnaire and ALSFRS-r and low score in ALSAQ-40 means better QoL and functional ability, more reverse correlation in comparisons would show greater validity. Spearman's rank correlation coefficient (Spearman's *ρ* or Spearman's Rho) was used to measure the validity.

## Results

The twenty-one enrolled patients were 27-77 years old (mean age = 58.7), with 52% females and 48% males. The duration of the disease was between 2-48 months and 52% of them were afforded to use Riluzole.

To evaluate the internal reliability, Cronbach's alpha coefficient was measured for each scale in the ALSAQ-40 in which alpha > 0.7 which means high reliability. Cronbach's alpha coefficient for different scales of ALSAQ-40 was 0.91-0.96 (Table 1).

**Table 1:** Cronbach's alpha coefficient demonstrating internal reliability of each scale in the Persian version of the ALSAQ-40

Scale	Order of questions	Cronbach's alpha coefficient
physical mobility	1-10	0.95
Activities of daily living	11-20	0.91
Eating and drinking	21-23	0.95
Communication	24-30	0.96
Emotional aspect	31-40	0.94

Item-total correlation was also used to evaluate the correlation of each question with total score in which item-total correlation greater than 0.4 means satisfactory correlation of each question with related scale. All the 40 questions in the questionnaire had correlation greater than 0.5.

Physical and mental health components from SF-36 were used to evaluate the validity of the related scales in the ALSAQ-40. Correlation coefficient (rho) was 0.771 for the physical aspect (P < 0.001) and 0.599 for the mental health (P < 0.001). ALSFRS-r was used to evaluate other related scales, and correlation coefficients (rho) of the related scales are illustrated in Table 2. P-value for all the related domains was lower than 0.001.

**Table 2:** Correlation coefficients of the related domains in the Persian version of the ALSAQ-40 and ALSFRS-r

	Drinking and eating	Communication	Activities of daily living	Physical mobility	Emotional
Speech	0.74	0.78	-	-	-
Salivation	-	0.62	-	-	-
Swallowing	0.76	0.73	-	-	-
Handwriting	-	-	0.77	-	-
Cutting food	-	0.86	0.76	-	-
Dressing and hygiene	-	-	0.73	-	-
Walking	-	-	-	0.78	-
Climbing stairs	-	-	-	0.78	-
Dyspnea	0.65	-	-	-	-
Orthopnea	0.74	0.62	-	-	-

## Discussion

ALS which is although known as a motor neuron disease, has considerable impacts on several aspects of patients and consequently deteriorates their QoL.^15^ There are several questionnaires for evaluating QoL, such as SF-36 that its Persian version is available and is used for ALS as well as non ALS patients.^6^ Generic questionnaires rarely address some specific problems in ALS patients such as impact of dysarthria on communication and swallowing impairments. Moreover, they mainly focus on items such as pain which is not a prominent complaint in ALS. We would either omit some aspects of QoL or should use multiple questionnaires that are not practical and cost-effective. That is why using specific questionnaire applies to ALS patients.

The ALSAQ-40 was developed specifically to use in patients with ALS and proved to be a valid questionnaire for the evaluation of their QoL.^16^ The primary version consisted of 78 items; however, the next studies showed that 40 questions evaluate main complaints of these patients sufficiently.^11^ In this study, the ALSAQ-40 was translated to a comprehensible Persian version and its internal reliability and validity was appraised.

Cronbach's alpha coefficient was used to evaluate the internal reliability. When the coefficient is greater than 0.7, it indicates high internal consistency of the questionnaire and correlation of questions in each scale. In the present study, Cronbach's alpha coefficient for all the five scales of the Persian version of the ALSAQ-40 was greater than 0.9 which means high internal reliability. In other studies designed to evaluate internal reliability of the original version of the ALSAQ-40, Cronbach's alpha coefficients were greater than 0.7 that are in consistent with the coefficients in the present study.^7, 10, 12^

To evaluate the validity of the Persian version of the ALSAQ-40, we used the Persian version of the SF-36 questionnaire as the gold standard for assessment of the physical and mental health aspects. Reverse correlation between the related domains was calculated (because higher SF-36 score and lower ALSAQ-40 score meant better heath condition). Correlation coefficient (rho) was 0.77 for the physical and 0.599 for the mental aspect that suggested satisfactory correlation of the related items in the two questionnaires. In a previous study done by Jenkinson et al., correlation coefficient of these two domains in SF-36 and the original version of the ALSAQ-40-was calculated to be 0.66 and 0.65 for the physical and mental aspects, respectively.^7^

Furthermore, we used ALSFRS-r to evaluate other aspects of the Persian version of the ALSAQ-40 and it was demonstrated that all the related domains in the two questionnaires had correlation coefficient greater than 0.7 which means high correlation and validity of the questions in these domains. The results are in consistent with the previous study demonstrating the correlation of the related domains in ALSFRS-r and the original version of the ALSAQ-40.^7^

## Conclusion

This study demonstrated that the Persian version of the ALSAQ-40 is a reliable and valid questionnaire to evaluate QoL in Iranian ALS patients and properly addresses all the five main domains of their QoL.
